# Early Citizen Science Action in Ethnobotany: The Case of the Folk Medicine Collection of Dr. Mihkel Ostrov in the Territory of Present-Day Estonia, 1891–1893

**DOI:** 10.3390/plants11030274

**Published:** 2022-01-20

**Authors:** Raivo Kalle, Andrea Pieroni, Ingvar Svanberg, Renata Sõukand

**Affiliations:** 1 University of Gastronomic Sciences, Piazza Vittorio Emanuele II 9, 12042 Pollenzo, Italy; a.pieroni@unisg.it; 2 Medical Analysis Department, Tishk International University, 100 Meter Street & Mosul Road, Erbil 44001, Iraq; 3 Institute for Russian and Eurasian Studies, Uppsala University, Box 514, SE-751 20 Uppsala, Sweden; ingvar.svanberg@ires.uu.se; 4 Department of Environmental Sciences, Informatics and Statistics, Ca’ Foscari University of Venice, Via Torino 155, Mestre, 30172 Venice, Italy; renata.soukand@unive.it

**Keywords:** history of ethnobotany, early citizen science studies, history of ethnomedicine, archive data, ethnopharmacology, plant identification

## Abstract

Presently, collecting data through citizen science (CS) is increasingly being used in botanical, zoological and other studies. However, until now, ethnobotanical studies have underused CS data collection methods. This study analyses the results of the appeal organized by the physician Dr. Mihkel Ostrov (1863–1940), which can be considered the first-ever internationally known systematic example of ethnopharmacological data collection involving citizens. We aim to understand what factors enhanced or diminished the success of the collaboration between Ostrov and the citizens of that time. The reliability of Ostrov’s collection was enhanced by the herbarium specimens (now missing) used in the identification of vernacular names. The collection describes the use of 65 species from 27 genera. The timing of its collection coincided with not only a national awakening and recently obtained high level of literacy but also the activation of civil society, people’s awareness of the need to collect folklore, the voluntary willingness of newspapers to provide publishing space and later to collect data, and the use of a survey method focusing on a narrow topic. While Ostrov’s only means of communication with the public was through newspapers, today, with electronic options, social media can also be used.

## 1. Introduction

Presently, a large number of wildlife observations in Europe are collected every year with the participation of volunteers, especially within ornithology. Amateurs have been of immense importance to many flora projects (national, provincial, and local floras) since the late nineteenth century and still are in many European countries. Based on this data collected through citizen science (CS), many distribution atlases (e.g., plants, mammals, and birds) are issued. Today, the importance of CS is growing exponentially, and it is already considered as an important pillar of science popularization and communication. For instance, on the initiative of the Swedish parliament in 1991, there is an on-going national inventory of all species of animals, vascular plants and mosses, fungi and lichens, and algae in order to map the biodiversity and this involves NGO’s, volunteers, and, in fact, anyone interested in nature in Sweden [1]. The inventory project (Artdatabanken = Swedish Species Gateway) is organised by the Swedish Species Information Centre at the Swedish Agricultural University, and they are working full-time on accumulating, analysing, and disseminating gathered information concerning the species and habitats occurring in Sweden. Citizen science is an important part of this project. Similar projects are underway in many other countries cf. [2], for example, in 2008, the Estonian Environmental Board started collecting people’s wildlife observations, which thousands of individuals submit every year (see https://lva.keskkonnainfo.ee/ accessed on 21 December 2021). Citizen science is also receiving increasing attention as a mean of collecting indigenous and local knowledge to better manage and conserve ecosystems [3]. For example, an initiative was launched in Spain in 2017 where non-scientists are able to add traditional ecological knowledge to the electronic database CONECT (www.conecte.es accessed on 15 January 2022) [4,5].

However, after almost twenty years of existence as a concept, the definition of CS still depends on the context [6]. Therefore, we are inclined to view this method of involving citizens in science as something new, but history tends to repeat itself, as human nature does not change as quickly as the current technological progress would allow us to assume.

The roots of CS are sometimes traced as far back as Aristotle, while some refer to Swedish botanist Carl Linnaeus (1707–1778) or the amateur documentation of the flowering of cherry trees in Japan [7,8]. If today the CS method is primarily related to the supplementation of institutional (e.g., research, memory, or state institutions) collections, then in the 18th and 19th centuries, methods similar to what we assume in some contexts to belong to CS were used to supplement private collections. To record folk knowledge among the peasantry, the Sami, local healers, etc., was the normal process for Linnaeus and his pupils. The reason for this was mainly economic and to make botany useful. Why import expensive medicinal plants from abroad when equivalent plants could be found among the local inhabitants? Why import dye plants when the peasantry already successfully used lichens and plants to colour their fabrics? Why not increase the local authorities’ knowledge of plants that could be used as a flour substitute in times of bad harvest? Linnaeus developed a research program with a questionnaire for travelling scholars [9] that they could use to gather local knowledge about these things. His own travels in the 1740s were expeditions sponsored by the government to record knowledge about wild plants and lichens that could be used as medicinal plants, dye plants, and food plants [10]. The first professorship in national economy was actually held by botanists and there were many publications dealing with the economic aspects of plants. For Linnaeus, this was an important way to show the usefulness of botanical knowledge for the university and the authorities, and for his many followers this knowledge was a way to improve the economy of Swedish communities [11]. In Sweden, this data collection included hundreds of locals, such as vicars, physicians, pharmacists, noble landowners, bailiffs, and even curious peasants, who participated in collecting plants and sending information. Linnaeus himself had around 600 correspondents, not only in Sweden (including Finland) but all over the world, who provided him with plants and information on the use of plants and animals cf. [12,13].

Some of these correspondents (for instance, Pehr Kalm in Finland; Johan Peter Falck, Johann Gottlieb Georgi, Johan Georg Gmelin, and Peter Simon Pallas in Russia) had their own networks of locals who gathered data for them [14]. One of these correspondents was the Norwegian Bishop Johan Ernst Gunnerus (1718–1773), who, very much inspired by Linnaeus, for his project on the Flora Norvegica (published in Latin in two volumes in 1766 and 1776) involved a wide network of clerks who collected information and plant specimens from all over Norway [15]. In Italy, Vincenzo de Romita (1838–1914) used a worldwide network of voluntary professionals to supplement his private collection with wildlife exhibits and data and was one of the first to use such an approach [16]. De Romita’s nature collection is located today in a museum bearing his name (http://www.centrostudideromita.it/ accessed on 21 December 2021).

Yet, the essence of CS, as it is perceived today, is the thoughtful participation of citizens with no specific science-related training in a joint effort to better understand a particular scientific or social phenomenon, often from an interdisciplinary perspective [17]. A more recent and thoroughly researched example is that of the publication of questionnaires by a botanist from Jagiellonian University, Józef Rostafiński (1850–1928), which, mainly in 1883, attracted numerous correspondents who sent herbarium specimens, some of which are still preserved, along with their responses regarding the use of wild food plants [18,19]. Indeed, from the middle of the 19th century, influenced by romantic nationalism, many early (then often amateur) folklorists in Nordic and Central-European countries started campaigns to collect lyric and practical folklore through newspaper advertisements calling on people to contribute. For example, the “first appeal to initiate the collection of Swedish-speaking folklore appeared in the Vaasa-based newspaper Ilmarinen on 5 April 1848”, although without success [20]. However, many diverse calls yielded a significant number of collected records, supported by intensified peasant education [21], and those campaigns had a strong component of CS. In 19th-century northern Europe, ethnobotany, the specific aspect of local practices that are of interest to us, was considered a grey area between folklore and the natural sciences (it was not until 1895 that John Harshberger coined the term ethnobotany). The majority of those numerous works collected in the 19th century were buried under the tons of never thoroughly analysed folklore, and for a valid reason: in folkloristic texts the local name itself, due to its ambiguity and fluidity, is rarely sufficient to make a direct connection between the botanical plant taxa and its use. In Europe, the 20th century was more productive in terms of plant-lore collection (including the involvement of students in the collecting work, as is seen in references to collections from the 1930s in Estonia [22] or Ireland [23]). Such early ethnobotanical collections are still to be explored and analysed, not only for the reported plants and uses, but also for the methodology with respect to the successful involvement of citizens in the preparation of a scientific study.

### 1.1. Mihkel Ostrov and Ethnopharmacology in Estonia the 19th Century

An excellent study ground for the history of CS in ethnopharmacology is provided by the collection of Estonian medical student and later doctor Mihkel Ostrov (1863–1940), who gathered information for a few years beginning in 1891. Through newspaper appeals, following the example of folklore collectors of his time, he succeeded in amassing a remarkable number of responses, including herbarium specimens. Although the original samples were not preserved, we still have the interpretations provided by Ostrov, along with his identifications of the plants. Ostrov, although studying to be a medical doctor, was very open to people’s use of plants, as he had experience collecting them himself as a mentee of the pastor and folklorist Jakob Hurt (1839–1906).

Before Ostrov, Estonian ethnopharmacology had already been documented with proper botanical identification but only in very specific locations. It was first documented by the Baltic German doctor Johann Wilhelm Ludwig von Luce (1756–1842), who published the results of his research in a work titled “Heilmittel der Ehsten auf der Insel Oesel” [24], which can now be considered one of the first local pharmacological works in Europe, and it was documented a few years later, in 1831, by the amateur botanist and pastor Johann Heinrich Rosenplänter (1782–1846), who wrote a manuscript that remained unpublished [25]. They were both Baltic Germans and Estophiles, yet the Estonian language was not their mother tongue. Unlike Rosenplänter and von Luce, Ostrov shared the same cultural code and language with the people who sent him the information. Neither Rosenplänter nor von Luce used a CS approach *sensu stricto*, as they questioned people and collected plants themselves (although being amateur scientists), without using mediators, as far as we know. Ostrov, however, received a large part of his information through a network of correspondents, which is comparable to the current methods of data collection employed in CS.

The national awakening of Estonians, which began in the middle of the 19th century, reached its peak at the end of that century. This was accompanied by the emphasizing of the value of “antiquities” and, above all, the collection of lyrical folklore. The first attempt to involve the wider population in the collection of “antiquities” was made by the pastor and folklorist Matthias Johann Eisen (1857–1934). Starting in 1883, he began to make thematic appeals in newspapers. He was particularly active in putting forward calls to the general population from 1887. His interest was initially in fairy tales and later widened to general “antiquities” [26].

The largest action to involve the general public was the call made in 1888 by Jakob Hurt: “A couple of requests to Estonia’s most alert sons and daughters”. In almost 18 years, nearly 1400 correspondents sent him data. Hurt appealed to his collaborators, asking if they could, in addition to providing their knowledge, question people living in their village. Hurt’s emphasis was also on the old and the archaic, and especially on lyrical folklore. Everyday practices, where herbal treatments belonged in those days, were not of great interest, and the methods selected for collection were not adequate. Although dedicating a special chapter to plant use, Hurt classified herbal treatments as “beliefs and customs”. Hurt’s attitude and classification also gave direction to all other subsequent folklore researchers [27,28].

Jakob Hurt regularly reported through newspapers the sources and quantity of information and what kind of folklore was sent to him. In addition to the reports, he also made repeated calls to encourage new collectors. In total, Hurt published more than 150 reports during his lifetime.

Before the appeals, Jakob Hurt had been collecting folklore privately for about ten years. He also used scholarships for this purpose. At the end of 1886, he turned to the Estonian Students’ Association (EÜS) for help in collecting folklore. The society discussed it at the beginning of 1887, and among the first to agree to do fieldwork was a medical student at Dorpat (Tartu) University named Mihkel Ostrov. Hurt also sent a very comprehensive collection guide in German titled: “Bemerkungen zur Richtschnur beim Samm[e]ln alter estnischer Volkslieder, Märchen, Sprichwörter, Sagen etc”. Since Hurt’s home and school language was German, it was probably more convenient for him to make a guide in that language. Hurt promised to cover the fieldwork costs and salary of the scholarship holders. In 1887, Ostrov and his companion Oskar Kallas (later an Estonian diplomat and folklorist, 1868–1946) collected a very rich sample of folklore in the parishes of Laiuse, Torma, Simuna, and Põltsamaa (central Estonia) [29]. Ostrov also conducted expeditions on behalf of Jakob Hurt in Alutaguse (north-eastern Estonia) in 1888 and in Läänemaa (north-western Estonia) in 1889, where he also collected valuable material. In 1890, however, he went alone to Läänemaa to collect folklore. While collecting in Läänemaa, he also wrote down the first use of medicinal plants: “From the flowers of *liivatee* (*Thymus serpyllum*) they make a medicine against coughs and lung diseases” (EKS, c, page 63). The second impetus for Ostrov’s personal collection was certainly that he was a member of the most progressive society of his time, the Society of Estonian Literati (active from 1871 to 1893), which was actively involved in collecting “antiques”. From 1892 to 1893, Ostrov was also a board member of that society.

**Figure 1:**
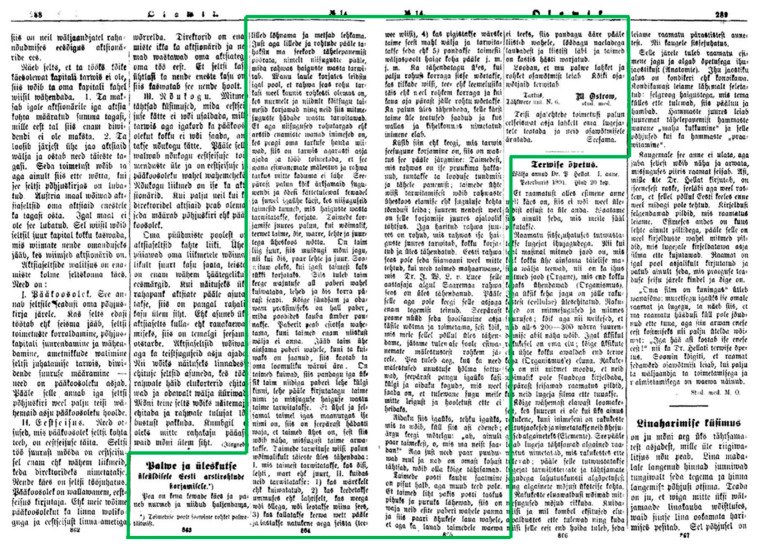
Ostrov’s first survey plan and its placement on two pages in a newspaper (Olewik, 1891 April 8, No. 14).

Ostrov therefore already had extensive experience in the field of folklore. Apparently because of this, he made his first public appeal while still a student (he graduated in the second half of 1891) on 6 April 1891, in the newspaper Postimees. At the same time, Jakob Hurt was communicating his appeals through the same newspaper. Ostrov states in the call, “When I collected old songs among the people, I saw that there are still many folk healers everywhere who collected a lot of medicinal plants from nature and use them to treat many diseases” [30]. His early ambition is clearly shown by the title of the appeal, which aims to gather “general information about Estonian folk medicines”. The newspaper, for its part, added a request for active participation and for other newspapers to publish the call as well. This was what the newspapers Olevik [31] (see Figure 1) and Sakala [32] did on 8 and 26 April respectively. His call was scheduled for early spring so that collectors could pick the first plants in spring and summer. The call contained detailed instructions on how to pick and dry the plants and how to send them by post in a wooden box so that they would not be damaged. He also asked participants to write in detail how these plants were used and for what purpose, as well as their popular names. It is worth noting that, in the same newspaper, Ostrov also gave a positive review of the first Estonian medical textbook [33].

For feedback, Ostrov employed the same methods as did his mentor Hurt: in July of the same year, he published the first report [34]. At the end of the report, Ostrov said that medicines made from stone and animal products could also be sent. In addition, he asked for detailed descriptions of the diseases and their symptoms, as popular names were ambiguous. The report was again re-published in other newspapers [35]. The second report was published in October [36,37].

Ostrov made his next call in April 1892, again in the spring so that people would have time to prepare for plant collection. As with the first appeal, he began his call with a positive description of nature and an inspiring tone: “Winter is over, spring is here, buds in the tree, plants are emerging. Now the picking and collecting, which went into hibernation when autumn arrived, can come back to life again, and the work in progress can now be carried forward”. The summons stated that the newspaper (Postimees, Sakala, and Olewik) editors agreed to accept herbarium samples and then send them to Ostrov [38,39,40]. The reason may be that in the intervening time he changed his place of residence and got a job in Nõo parish as a rural municipality doctor. The third report was not published until late autumn, and, for some reason, it blamed the “sad summer” for the modest collection activity without specifying what that means. “Last sad summer also seems to have had a detrimental effect on the collection of folk medicines, as this summer’s collection is only a quarter of the last” [41,42]. The third and final call came on April 26, 1893 [43]. There, too, Ostrov announced that packages with plants should be sent to the newspaper’s editorial office because his residence is not permanent. However, the newspaper editors kindly allowed the samples to be sent to them. Ostrov’s collection work was likely interrupted as a result of his constant changes in residence: from 1892 to 1893 he worked in Nõo; in 1893 he went to Russia to work, first to Smolensk, then in 1895 to Pskov; and in 1898 to Jelgava, present-day Latvia, where he worked as a railway doctor on the Moscow-Ventspils railway line. From 1914 to 1919, Ostrov worked as a military doctor in World War I and later in the Estonian War of Independence. At that time, he became the Commander of the Estonian Army Health Care Government and earned the rank of Sanitary Major General of the Sanitary Service. Later, he was employed as a school doctor in Põltsamaa, and in 1927 he retired [44].

### 1.2. Work’s Aim

The main aim of this work is to understand the reasons for the effectiveness or ineffectiveness of the earliest citizen science methodology in ethnobotany. To that end, we:

1. analyse the traditional medicinal plant use of 19th-century Estonia, and
2. compare it, to the extent possible, with folklore data from the same period, based on earlier publications (Jēkabs Alksnis 1894 [45] and Johann Wilhelm Ludwig von Luce 1829 [24]), manuscripts (Johann Heinrich Rosenplänter 1830s [25]), and the information contained in the ethnomedicine and ethnobotany database HERBA (19th–20th centuries) [46].

We expect to observe some specific plant uses which will not be detected in HERBA due to the ambiguity of some plant names.

## 2. Results

### 2.1. Correspondents and Their Contributions

**Figure 2:**
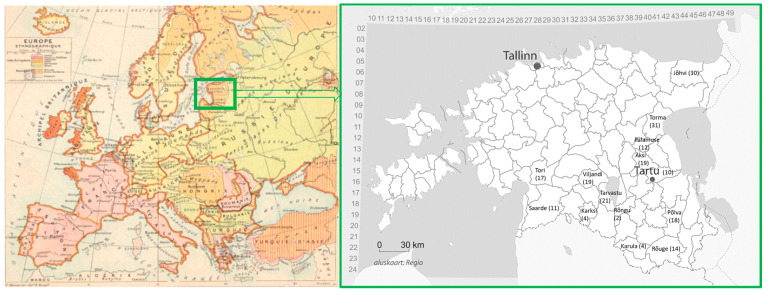
Historical map (“Atlas Melin Historique et Geografique” published by Andre, Paris, 1900) of the region and the parishes from which Ostrov received correspondences regarding plant uses (UR). Parish division of the territory of present-day Estonia at the end of 19th century.

Sixteen people were identified as correspondents of Mihkel Ostrov (Table 1). The correspondents originated from 14 parishes (Figure 2), yet there is a mismatch between the parishes mentioned in the reports and those indicated as the origin of the knowledge in the manuscript. The parish having uses (18 use reports (UR)) but no correspondent was Põlva. There is also a discrepancy between the number of plants mentioned in the reports and the UR from a specific parish; for example, in the reports Ostrov mentions that he received 36 plants from Rõuge’s only correspondent, J. Orraw, yet in the manuscript we were able to find only 14 UR and 8 plant taxa.

With his calls, Ostrov succeeded in mobilizing the active part of society most likely also interested in medicinal plants. Among his correspondents there were several practicing doctors and students of medicine, who probably knew him from either school or the Estonian Student Society, which included all active students of that time. One of Ostrov’s correspondents, Dr. Henrik Koppel, was, from 1920 to 1928, twice elected rector of Tartu University, yet at the time of correspondence he had just recently acquired his medical diploma and was preparing to defend his doctorate. Henrik Koppel was collecting folklore for Hurt while still in high school and eventually he married Hurt’s niece, Sophie.

The list of correspondents also contains several farmers of whom not much is known, as well as local activists (e.g., members of charities and folk choirs, etc.) and intellectuals. Ostrov indicated that several collectors obtained data through interviewing villagers. One of them, Hans Kosesson, who worked as an assistant vodka master at Tarvastu Manor, interviewed nearly ten manor serevants, whose names were provided by Ostrov in his manuscript (EKS, c, page 34).

There were also three women included among Ostrov’s correspondents. Although women were only allowed to officially study at the university in 1915, in village schools girls received an education equal to that of boys. All three of the young women that contributed to Ostrov’s collection were socially active and contributed in diverse ways to the development of Estonian culture. Elise Aun and Helene Maasen knew each other through society work [47] and it is very likely that they also knew Elise Torim, the third female correspondent. Helene Maasen, who studied at a private German-language school and later also translated foreign language texts, was Hurt’s most important female correspondent and one of the best correspondents overall. Elise Maria Torim also collected folk songs for Hurt but did not consider them polite enough to submit; these songs were eventually sent to Hurt by Ostrov himself (H II 25, pages 1097–1134). After at least four years of acquaintance, which also included responding to Ostrov’s call in 1891, Elise and Mihkel married in the fall of 1893.

Since Hurt and Eisen also collected at the same time, half of Ostrov’s correspondents were also active collaborators of those researchers. Of these correspondents (Table 1), four also reported plant uses or medicinal magic to Hurt and one did so to Eisen; however, the reports found in other collections rarely overlap with the ones reported in Ostrov’s manuscript. As the source of the information is not traceable in Ostrov’s report, the exact proportion of overlap cannot be determined.

### 2.2. The Ethnopharmacology of Ostrov’s Collection

Ostrov’s collection comprises 65 taxa, of which 64 were identified on the species level and one (*Betula*) on the genus level, belonging to 27 families (Table 2). The most widely used taxon was *Achillea millefolium* (with 20 UR), followed by *Plantago major* (15 UR), and *Valeriana officinalis* (10 UR). The most commonly used family was Asteraceae (12 taxa and 53 UR), followed by Lamiaceae (5 taxa and 18 UR), Valerianaceae (2 taxa and 17 UR), and Apiaceae (4 taxa and 12 UR).

Of the 219 UR, the most mentioned uses were treatments for skin diseases (56 UR), followed by general and unspecified diseases (35 UR), diseases related to the digestive tract (33 UR), and respiratory diseases (29 UR). Among the general and unspecified diseases, the most prevalent were, at that historical moment, specifically defined culture-bound diseases, such as *halltõbi* (now interpreted as malaria; it had a well-defined set of cultural rituals in folklore), *pistja* (which is some kind of sharp pain of unknown origin inside the body, often treated by poking someone with or digesting something sharp), *rabandus* (now related to stroke, but at that time considered a suddenly occurring disease brought on by the wind), and *seesthaigus* (a kind of internal pain of unknown origin). Another fairly common use (7 UR) was the symptomatic treatment of tuberculosis, which was sometimes also called *rinnahaigus* (10 UR, lung disease refers to any disease of the lungs and could also include pneumonia and severe cough). Remarkably, tuberculosis was treated with a different set of plants than was *rinnahaigus*: the only overlapping taxon was *Polygala amarella*. Cough as a treated symptom was mentioned in 11 UR, with *Achillea millefolium* being the most mentioned taxon (3 UR); this taxon was also mentioned twice in relation to the treatment of colds (*külmetus*). Still very common among musculoskeletal diseases was the treatment of a kind of rheumatic disease, called *jooksva*, which referred to the “running” of the disease, as the pain often changed places. It was historically treated by plants “running” on the ground, many of them with names referring to the disease: *jooksvarohud/joosvarohud* [48]. Among skin diseases, the most prevalent ailment was boils (*paised*, 14 UR), for which *Tussilago farfara* (4 UR) was most often used; also in this group, a culture-bound disease *maa-alused* (different forms of urticaria; literally “from underground”) was frequently mentioned (6 UR).

### 2.3. The Disease Reflected in the Name of Plant

The variety of names reflected in Ostrov’s collection is extraordinarily high and they often refer to the disease or the symptom the plant was thought to heal. For example, all the taxa used to treat *maa-alused* had similar names, such as *maa-aluse rohi*, *mailaseroht*, *maalesehein*, *maaleserohi*, and *maavits*; *rinnahaigus* had the name *rinnatee* (literally “tea for lung”); chills from malaria were treated with *külmaväristuse rohud* and *värisejahein* (literally “plant against chills” and “trembling plant”). Bleeding was stopped almost exclusively by *verihein* (literally “blood grass”, 4 out of 5 UR, the remaining being a linen cloth). With a few exceptions, plants that have more than one UR also have more than one name. The exceptions included *Tussilago farfara* (called *paiseleht*, boil leaf, and used predominantly against boils), *Taraxacum officinale*, *Menyanthes trifoliata*, trees, and a few other culturally important taxa.

### 2.4. Comparison with Earlier and Later Sources

In comparison with historical sources, we found only a limited overlap of the taxa used with Rosenplänter’s collection [25] (Table 2). Only three taxa had the same use and name (*Veronica officinalis, Solanum dulcamara*, and *Briza media*), while two taxa had one similar use among many (*Menyanthes trifoliata* and *Plantago major*). Five more taxa are present in both sources, yet the uses do not overlap. The reason for this is that peasants used to have strict movement restrictions due to serfdom. Therefore, there was very limited interpersonal exchange or mixing of knowledge, including on the use of plants. It was not until the 1860s that peasants were allowed to move outside their parish, and in 1868 the last form of slavery was banned (the obligation of peasants to work for landowners) [49].

Compared with von Luce [24] there are twelve taxa for which some of their uses overlap (such as the application of *Tussilago farfara, Achillea millefolium*, and *Plantago major* on wounds, the use of *Viola tricolor* against eczema, and *Valeriana officialis* during childbirth). Some applications or preparation modes differ slightly, such as in case of *Carum carvi* and *Levisticum officinale*. Six jointly named taxa had different uses.

There are considerably more similarities with medical student Jēkabs Alksnis’s (1870–1957) article [45]: only slightly more than one third (23) of the taxa mentioned by Ostrov are not listed among those medicinally used in Alksnis. However, the majority of the overlapping taxa were either used for other aliments (26 taxa) or their exact use was not specified (2 taxa). For the remaining taxa, the uses overlap either fully (in a few cases) or partially. For some uses, the difference may be on the level of the details provided by the author; for example, sour cabbage (*Brassica oleracea*) was put on the head to treat headaches in both sources, yet Alksnis mentions boiled or fresh cabbage leaves, as whole cabbages were fermented, while Ostrov’s text lacks such information. Notably, while Alksnis describes the external use of the highly toxic plant *Cicuta virosa* L., he provides very few details of its preparation, while Ostrov’s manuscript describes four external uses, none of which overlaps with the other limited comments in HERBA [46], but it does overlap with the one use in Alksnis (tumor).

The similarity to the rest of HERBA [46] is quite considerable, although some of the overlaps may have several potential identification options. There were only two taxa (*Viola canina* L. and *Rosa canina* s.l.) found solely in Ostrov’s collection, and this may be due to the exact identification.

### 2.5. The Importance of Rituals

From the report it is also clear that there were some uses where the plant species did not matter, as some of them relate to the product obtained from the plant or some other features of the plant beyond its taxon. For example, it was not specified from which tree, either *Picea abies* or *Pinus sylvestris*, resin or tar, which was applied to wounds, was extracted. It was either applied by itself or as an ointment of melted resin with salt or lard. It was also irrelevant which taxon of tree, broken by thunder, was used for toothache. A piece of wood was broken from this tree and used to pierce the tooth.

Therefore, it seems that in the majority of those few cases where a ritual was involved, the ritual was more essential than the taxa used; for example, *koeranael* (literally “dog nail”, translated as boil/furuncle) was treated by going to the forest and breaking a stick from three trees to press the affected area. These sticks must then be returned to the same trees.

In 1888, outside of his collection, through his personal field experience, Ostrov wrote down three detailed treatment rites that were performed in the sauna room (in a village by the Narva River in Vaivara Parish). All of them were related to paediatric diseases whose origins were thought to be magical:

*A child has a worm defect when he turns and twists his head and rotates his eyes. This defect was treated as follows: two worm trees, [the ones] with which the worm was killed, were brought from the forest. One was put on the fire in the sauna oven and burned. When the oven was hot, another worm wood tree and a rope were placed on the sauna stove and covered with steam. Inside this steam, the child was whisked, which healed the mistake.*

(EKS, c, page 53)

*Dog disease is a disease of young children when they have a big stomach, a loose body, eat and drink a lot; but it dries out more and more in the warm weather, it does not kill. The disease is treated as follows: on three Thursday nights, two widowed women have to whisk a child and a dog in the sauna, one for the child, the other for the dog. Before whisking, the dog is washed in water, then the washing water is used for whisking, and the child is given [the same water] to drink. The one who holds the dog asks the whisker, “What are you whisking?” “I’m whisking dog disease!” “Whisk so that he will be healed!”.*

(EKS, c, page 52)

*The crying of young children at night is called “Öö itk” [cry of the night]. It is treated as follows: one old rope is put in water and a child is whisked with that water; the whisked baby is pulled 3 times through the rope twist; the water was poured out, and the rope is put back on.*

(EKS, c, page 52)

The sauna has historically been a very important place for treatment for Estonians. “Whisking” is the rhythmic hitting of yourself or other people with a bunch of twigs (a “whisk”), usually birch leaf twigs, in a hot steam room in the sauna. Its purpose is to massage and make the body sweat. In addition, the birch whisk used in the sauna has been considered therapeutic. This is also reflected in the texts sent to Ostrov. For example: “Whisking in the sauna with a whisk which is made from *emanõgesed* [“female nettles” *Lamium album*] makes infertile women fertile” (EKS c, page 64). This text combines the sauna as a ritual place and a symbolic plant name. Whisking in the sauna was used to treat various skin diseases and itching. Abscesses were also treated in the sauna: after whisking, wet wood ash was put on the abscesses and covered with a linen cloth. Birch leaves from the sauna were also used in ointments for skin diseases. Sleeping on a pillow of birch leaves, however, helped with headaches. Ritual whisking was performed when a child had various childhood illnesses.

According to Ostrov’s correspondents, birch was used for treating different forms of urticaria (called *maa-alused*): a cross was drawn on birch bark and then tied on the skin for three days, or a pentagon was drawn, pressed three times, and thrown into the oven. Birch sticks were also used to heal warts by pressing them with salt. After that, the sticks were placed at a crossroads and left there without looking back.

The texts of Ostrov’s correspondents show that other trees, such as alder (*Alnus* sp.), rowan (*Sorbus aucuparia*), and juniper (*Juniperus communis*), have been used in rituals as well. Of them, rowan and juniper have been considered sacred, because it was widely believed that their berries were marked by Jesus, an idea which was spread by the Evangelical Brotherhood and radical Christian congregations in the 18th–19th centuries. Although the first missionaries of the Evangelical Brotherhood arrived in Estonia and Livonia as early as 1720 and their activities began in the 1740s, this movement remained limited to Western Estonia and Saaremaa. It was not until the “granting of mercy” to the religious movement by Emperor Alexander I in 1817 (before that the free religious movement was forbidden in the Russian Empire) that the “new awakening” of the Evangelical Brotherhood and radical Christian congregations started to spread in the region. This became the “new time of awakening” of the Christian movement there. It is believed that at that time the so-called real Christianization and the abandonment of pagan customs also took place in Estonia and Livonia governorates. By the beginning of the 20th century, however, these religious movements had already lost their importance [50]. This is the reason why key earlier pagan customs and sacraments became associated with Christianity (such as trees or flowers, which were considered sacred). Such activities helped to bring Christianity closer to the local people.

Juniper was considered a tree of health and was used in the magic of controlling diseases. At the same time, the alder tree was important as a source to which a disease was transmitted. For example, one had to get rid of malaria by walking around the alder tree three times and each time exhale into a hole made in the tree, and then block the opening with a rowan tree (Hans Kosesson, H I 3, 312 (26)). In another example, nine pieces of alder tree on which to mark crosses were brought home from the forest and then swellings were pressed with them, after which the swellings disappeared (Hans Kosesson, H I 3, 266 (7)).

The folk calendar was also a part of the ritual. Ostrov’s correspondents mention Midsummer’s Day, June 24, as the anniversary of the folk calendar. Herbs harvested before or during this time were the best for treatment (H. Karu, EKS c, page 56). The plant species was not very important: all the blooming flowers harvested in the forest that day were used for healing. As Hurt had a separate question regarding the folk calendar, he received more of such information from Ostrov’s correspondents. Plants were also used for divination that day: single girls picked nine flowering plants on Midsummer’s Eve, braided them into a wreath, and laid the wreath under their pillows. Then a girl had to dream of her future husband (Helene Maasen, H III 8, 736 (10)). In the Midsummer evening, a special flower, *Erigeron acer*, was brought home. If at night your closed flowers are opened in the morning, then the next year will be good, but if it is still closed, then death is expected.

## 3. Discussion

### 3.1. Ostrov’s Report as a Cross-Section of Plant Use during the Time of Collection

The little overlap with Rosenplänter’s collection is not surprising, given both the temporal and geographical differences. However, the comparatively higher similarity with von Luce [23], a contemporary of Rosenplänter, is somewhat unexpected, as von Luce reported uses from a relatively isolated island. It may be that von Luce, himself, had already influenced some uses which he recorded as local. It can also be that that there were uses influenced by his predecessors or landlords that originated from scholarly medicine of that time. Overlapping taxa included *Achillea millefolium, Allium cepa, Artemisia absinthium, Matricaria chamomilla, Nicotiana rustica, Taraxacum officinale, Valeriana officinalis*, and *Verbascum thapsus,* although some of them were used for different purposes. All eight of these plants also overlapped with the ones listed by Jēkabs Alksnis [45] in a report published a few years after Ostrov’s collecting work. We can also detect the possible presence of *Arnica montana*, widely popularized in 19th-century media [51], although the name *arnikas* was associated, symptomatically, with *Leucanthemum vulgare*. Both von Luce and Alksnis were doctors, sharing a similar medical education background. While comparing with Alksnis [45], it is important to keep in mind that future Latvian professor of medicine Jēkabs Alksnis studied medicine at Dorpat (Tartu) University from 1890 until 1895 and was familiar, not only with Mihkel Ostrov but with his collecting works, which might have even inspired him. Moreover, Alksnis made a call through the newspaper, but not much about the results is known. He specifically named one doctor who was the only one who sent him properly dried plants [45].

An interesting example is that of non-native *Matricaria chamomilla*, whose uses were still unspecified in both von Luce’s report and Rosenplänter’s collection and were poorly represented with different applications in Alksnis, yet widespread with overlapping uses in later folklore. The reason for this was that in the middle of the 19th century a so-called “reading revolution” took place among the Estonian-speaking population, where a large number of peasants learned to read and popular books on the natural sciences were published [52]. As very few books on nature and its uses had been published in Estonian before the end of the 19th century, the newly published books were primarily translated from German. This is also shown by the subsequently popular names of chamomile such as “German flower”, “Germany Anthemis”, or “German dog daisy”.

The overlap of both the list of plants and their uses with HERBA [46] was even higher than expected. A major part of the information comprising the database was collected from slightly before (from 1886) to up to one century after Ostrov’s collection, while the collecting methods and objectives (focusing on the “old times”) remained the same. This overlapping and the presence of widespread uses and similarities detectable on the basis of names, shows that Ostrov actually received a good cross-section of the medicinal plant use of that period and raises the credibility of the method he used for that time. The methodology used by Ostrov, however, allowed for the more exact documentation of the taxa behind local plant names, and, thus, will allow better identification/interpretation of information collected later.

### 3.2. The Problems of Citizen Science Ethnobotany in the 19th Century: Ostrov’s Shortcomings

Ostrov used a method which was employed by other folklore collectors: calls in national newspapers. Thus, Ostrov’s data collection was public. He made calls to people, instructing them, and motivating their assistance by providing feedback. A drawback of Ostrov was that he did not have a fixed institution and often changed his residence. Communication went through the newspaper’s editorial office. So, unfortunately, he had no direct personal contact with people. Thus, misunderstandings may have arisen. For example, during the 4th Estonian General Song Festival (15–17 June 1891, in Tartu), someone left Ostrov a package containing dried plants and their uses at the Estonian Literary Society. However, Ostrov said that the package had disappeared from the association and kindly asked his anonymous assistant to resend them the following year [36,37].

A comparison of the reports published by Ostrov in newspapers with his manuscript archive reveals a discrepancy. For example, the manuscript contains 18 plant uses from Põlva parish in 1891. However, none of newspaper reports contain any information about this parish. Moreover, Hurt and Eisen, the largest collectors, do not have any correspondent from that time providing ethnobotanical information on the use of plants from Põlva parish. The identity of the person who sent the fairly large collection is still unknown. It could have been a local pharmacist, doctor, or another village activist. Perhaps this person asked to remain anonymous so that their name would not be mentioned in the newspaper.

Both directly and indirectly, Ostrov’s medical data collection was influenced by the rivalry between the great collectors Hurt and Eisen. Hurt heavily lobbied for himself. He told the editor of the newspaper Olewik that he was the right person and the only one who could collect oral antiquities in Estonia. Hurt also wrote critically in the newspaper about Eisen’s collection and data analysis methodology. For this reason, the newspaper Olewik banned Eisen’s calls in 1893 (see [53]). Thus, Ostrov’s data collection may have been left unfinished due to a quarrel between the two largest collectors. The newspaper, which a few years before encouraged its readers to collaborate with Ostrov, had now changed direction.

### 3.3. Pioneering Methods in Ethnobotanical Data Collection

Ostrov’s collection method was innovative at the time. Although Hurt obtained a greater number of plant use records, the collection of Ostrov can be considered higher quality, as his plant identification is reliable. It was also the first successful attempt to collect folk medicinal knowledge accompanied by herbarium specimens in Estonia through an approach currently known as CS. Earlier, such an attempt was made by the German-born professor of pharmacy Johann Georg Noël Dragendorff (1836–1898), who in 1877 made a similar appeal, yet received no responses, most likely because his appeal was in German and therefore incomprehensible to potential correspondents [54,55]. As a general call to assist researchers, the active population or people interested in the subject took part. The more generalist collection of Hurt, which attracted more than a thousand correspondents, yielded results qualitatively comparable to the finely defined questionnaire of Ostrov that was answered by only a few dozen. This was due to activists working with both men. At the same time, it also shows that outside special interests (e.g., medics and pharmacists) ethnobotanical knowledge was known or noticed by only a limited number of people.

**Figure 3:**
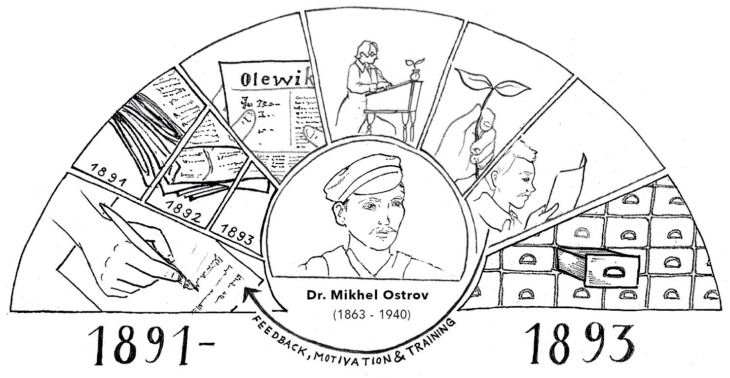
Visual representation of Mihkel Ostrov’s ethnobotanical data collection and communication with correspondents. (Credits: Johanna Lohrengel).

The success of the collection of Mihkel Ostrov (see Figure 3) is the result of a combination of several factors.

- The fertile ground previously prepared by the Estonian “time of awakening” and the ongoing collection of folklore by Jakob Hurt. The enthusiasm of the correspondents and the extent of their contribution was fuelled by the general understanding of the need for the preservation of “antiquities”.
- He knew what he was asking for. Having had field experience, Ostrov knew exactly what to ask and how to get people interested in the subject.
- With the call, Ostrov gave his correspondents something in exchange. He taught, in great detail, how to collect and press plants in order for them to be safely preserved. This kind of instruction may have been much appreciated.
- At the end of the 19th century and the beginning of the 20th century, the image of science and the scientist was different than it is today. For ordinary people at that time, a scientist could simply be a university-educated specialist. Moreover, the so-called researcher did not have to be working at a research institution. It was acceptable for people to give their data to a freelance scientist as well. The greatest disadvantage of a freelance researcher (with whom Ostrov could be grouped) was the lack of free time to analyse data. Therefore, the collected material waited for further researchers.
- Ostrov’s collection method showed that the “less is more” rule applies when employed correctly.
- Following the successful example of Jakob Hurt, Ostrov replied to all correspondents publicly, stressing the importance of their contribution and prompting them to send repeated responses, as well as assuring prospective correspondents that their work would also be acknowledged.

The time of collection of Mihkel Ostrov coincided with the rise of national awareness among Estonians, combined with a recently acquired high level of literacy (reaching over 90% by the end of the 19th century [56]) and a strong background in the basics of natural science taught at village schools [57] and through popular science literature [52]. It was a time when the active part of the peasantry and young intellectuals were searching for outlets in which to channel their energy and contribute to the development of the nation, but the majority of the various societies that later attracted them were not yet formed [58]. Ostrov motivated volunteers by appealing to this desire, namely by helping to halt the loss of traditional knowledge, and this remains one of the main motivations for cooperation today. Just as the vast majority of Ostrov’s volunteers were well educated, today a higher level of education increases volunteer involvement in the collection of traditions (cf. [4]).

It is important to note that at least three women took part in Ostrov’s calls. Thus, women were also involved and expected to contribute to social activity. Gender equality in basic literacy already existed at that time in the territory of present-day Estonia, although it was only in 1915 that the first woman was allowed to study in Tartu University.

## 4. Materials and Methods

### 4.1. Data

**Figure 4:**
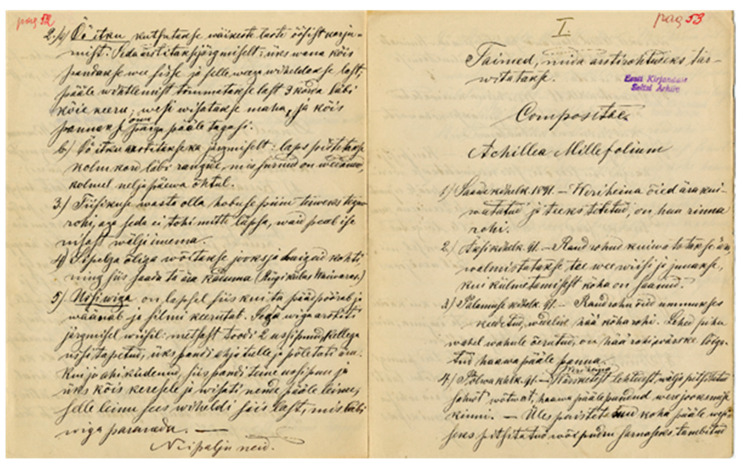
Sample of Ostrov’s manuscript in the Estonian Folklore Archives.

Ostrov’s collection is currently housed in the Estonian Folklore Archives, among the collections of the Estonian Literary Society (EKS), folder “c”. Folder “c” stores mails that were sent to the society between 1907 and 1917 and contain folk beliefs and folk medicine; Ostrov’s material can be found on pages 33 to 76 (EKS, c, 33–76, see Figure 4). The collection consists of three parts, each of which is sewn together with thread. Ostrov sent them from Jelgava to Tartu in three separate parcels. As Ostrov’s cover letters were not preserved, neither the year nor to whom he sent his collection is known. However, the scant remarks suggest that he knew the person intimately. Thus, it can be assumed that this person was his good acquaintance Oskar Kallas, who was one of the founders of the Estonian National Museum (ENM), established in 1909, and a member of the board of the EKS.

The Estonian Literary Society was founded in 1907 and Mihkel Ostrov and his wife immediately became members. This society became the most widespread and broad-based society, which included representatives from many walks of life. The aim of the society was to promote literature, science, and art in Estonia, as well as for members to get to know their country and people comprehensively and to make the results of completed work available to the public. After the October Revolution of 1917, the activities of the society stalled until the end of the Estonian War of Independence in 1920. In 1940, after the occupation of Estonia by the USSR, the society was disbanded, but it was re-formed in 1992. The Society issued the journal Eesti Kirjandus until 1940, and Ostrov became a journal contributor while in Jelgava. The journal also began to mediate the ENM’s calls to the general public to collect “antiquities” (ENM’s public calls to collect traditions continue to this day). However, there were also other calls, for example, in 1912 for the Estonian Students’ Society to collect folk plant names and the call of veterinarian Johannes Kool to collect folk animal treatments. It is not known which appeal Ostrov took part in and then sent his own collection to the society.

The Ostrov collection is well systematized. Latin binominal names based on herbarium specimens have been added and plants are classified by family. The first correspondence contains general folk medicine, beliefs, and prescriptions for herbal remedies, as well as information collected by him from 1888. In that letter, Ostrov used only the folk plant names by which we identified the species (see Table 2, where there is no Ostrov identification). The second letter contains a list of species of the family Asteraceae, based on Latin names and their uses. At the end of this letter there is a note stating that he will send the next list of plant families as soon as he can write them down. The third letter describes the following families and subfamilies [names unchanged]: Rosacea, Labiatae, Umbelliferae, Scrophulariaceae, Papilionaceae, Urticaceae, Solaneae, Polygaleae, Violaceae, Cruciferae, Valerianea, Plantaginea, Aroidea, Graminea, Boragineae, Ericaceae, Liliaceae, Gentianea, Orchidea, and Lichines. At the end of the third correspondence there is an indication that “The end will be with the next letter”. However, there is no end. Whether the absence of the next part was due to the disappearance of mail during difficult political times or the recruitment of Ostrov into World War I remains unknown today. Ostrov used the names of archaic families and subfamilies (e.g., Plantaginea (*Plantago major*), Valerianea (*Valeriana officinalis*), Lichines (*Cetraria islandica*), Aroidea (*Acorus calamus*), Gentianea (*Menyanthes trifoliata*), etc.), which were predominantly in circulation in the 19th century. Therefore, it can be assumed that Ostrov identified herbarium specimens as early as the end of the 19th century using a German-language plant reference book, because there were no reference books in Estonian at that time. According to recent data, *Archangelica officinalis* does not grow in Estonia. Ostrov therefore probably used a key book in which this plant was included. The folk name, *heinputk*, refers instead to the species *Angelica sylvestris*. In such cases, we used the popular name of the plant as a guide.

No herbarium specimens have survived to this day. Estonia’s first ethnobotanist Gustav Vilbaste (1885–1967) points out in his book that the collection of the EKS contains about a dozen dried plants. He thought that these may be plants that were sent to Ostrov [59]. The Estonian Folklore Archives indeed holds one small box in which herbarium samples of unknown origin are stored. It may be that the specimens in this box were brought to the archives by various collectors over decades, as there is no information on the time or origin of their collection. Therefore, we cannot determine if it contains plants sent to Ostrov, as we can no longer univocally connect the specific information on plant use with the sender (which also sets certain limits to the analysis).

The Latin plant names provided by Ostrov were adjusted to follow those listed in the Plants of the World Online [60] database and the European Flora [61] (these are presented in the “Taxa” column in Table 2); family assignments follow the Angiosperm Phylogeny Group IV [62].

### 4.2. Analysis and Comparisons

The names of the correspondents were provided in the three reports published in the newspaper Olewik [34,36,42]. Where possible, the first names and life dates were identified through bibliographic and biographical research. Identifications were complicated by the fact that only the initial of the first name, the surname, and the parish were known. Therefore, some people remained unidentified. The search was based on the biographical database of the analytical bibliography of the Estonian press (http://www2.kirmus.ee/biblioserver/ accessed on 21 December 2021) and biographical data from the central database of folklore collections (https://kivike.kirmus.ee/ accessed on 21 December 2021). Lastly, missing information was provided by Rein Saukas, a folklore historian who has thoroughly studied the biographies of the Estonian folklore correspondents, from his personal archive.

The data of Ostrov’s manuscript was transcribed and entered into a spreadsheet. The use report (UR) of each plant was calculated by summing its different uses. The UR of each correspondent was calculated by summing all plant uses. Use reports were separated and emic disease categories correlated, as much as possible, with the current International Classification of Primary Care, 2nd edition (ICPC-2, Updated March 2003). As such correlations were not univocally interpretable, they could only be carried out on the very general scale and should be viewed with caution. In order to bring the emic diseases into line with etic diseases, we used the chapter on folk medicine from Gustav Vilbaste’s book [59], which contains the Latin names of the diseases. We also used Vilbaste’s book and the HERBA [46] database to describe emic diseases. However, it is impossible to identify historical diseases retrospectively with 100% certainty. Therefore, we have also shown in Table 2 the original names of the disease in Estonian (or dialect) in the manuscript.

Therefore, the results were compared qualitatively with the content of HERBA [46]. The database HERBA contains texts on the medicinal use of plants reproduced from eight major collections of the Estonian Folklore Archives collected from 1860 to 1996 (for more details see [63]). Ostrov’s collection is also part of HERBA, and this was taken into account. As all archive texts in the HERBA database are coded with archival reference numbers, we separated out the texts of the Ostrov manuscript (EKS, c, 33–76) before analysis. The comparison was made from the standpoint of local plant names, taking into consideration the potential limitations of the absence of specimens for early historical texts. Qualitative comparison was made with the early historical uses recorded in Rosenplänter’s (Pärnu parish) manuscript [25] and the publications of von Luce [24] covering Saaremaa and Alksnis in Latvia [45] (using for last two digitalized database [64]).

## 5. Conclusions

Dr. Mihkel Ostrov succeeded in accumulating, with the help of about two dozen people, the largest private medicinal plant use collection from the end of the 19th century, whose credibility is enhanced by the fact that the plants were identified on the basis of herbarium specimens sent to him by his correspondents. Ostrov’s collection provides a cross-section of folk medicine and, to lesser extent, the healing rituals of herbal medicine at the end of the 19th century. It also shows the high diversity of both plants and uses known at that time in the territory of present-day Estonia, and its wealth increases the credibility of the rich medicinal plant folklore accumulated in Estonia since 1866.

The success of this CS endeavour was the result of a combination of several favourable factors, either in the environment of the time or created by the collector himself. The background factors, allowing the collection to happen at the right moment in the right place, included the recent abolishment of serfdom and access to education, the rise of national consciousness, the creation and activation of professional and student societies, and the simultaneous collection of general folklore. The factors added by Ostrov himself were the respectful approach towards his correspondents, providing specific guidelines and sharing knowledge, the public acknowledgment of their efforts, and, probably, his personal influence (as his correspondents included some of his classmates and his wife to be). The result was a fruitful and mutually beneficial collaboration where the contributions of citizens were publicly acknowledged, an approach that should serve as a good example for CS today. The collecting methods of Ostrov were pioneering for the time, yet they are still applicable from the viewpoint of modern CS. This work indicates that CS in ethnopharmacology in its wider meaning known today was born with studies of this kind, having its roots in the folklore collections of the 19th century in northern Europe.

We hope that lessons from the past can offer the modern scientist a good foundation for the development of the future involvement of citizens in studying ethnobotany.

## Figures and Tables

**Table 1:** The correspondents of Ostrov, based on the reports published in newspapers. Correspondents of Ostrov who also sent medicinal data to Jakob Hurt (H) or Matthias Johann Eisen (E) and the references in the respective collections.

Name and Life Dates of Correspondents	Profession	Parish	First Report (1891a)	Second Report (1891b)	Third Report (1892)	Non-Ostrov Archive Reference and Location of Correspondence (Range of Pages/Year)
Peeter Metusala (1869–1950)	Tailor	Karula	7 medicines			
J. Orraw ^1^ (?–?)	Farmer and potter	Rõuge	24 plants and 1 medicine	12 plants and 1 medicine		
Andres Saal (1861–1931)	Teacher, writer, and journalist	Tori	16 plants and 6 medicines			H II 21, pages: 9–64/1888
Elise Torim (1868–1929) (F)	Later wife of Mihkel Ostrov	Äksi	9 plants	14 plants		
Henrik (Heinrich) Koppel (1863–1944)	Doctor and later medical researcher, lecturer, and rector of the University of Tartu	Otepää	24 plants			
H. Karu ^3^ (?–?)	Farmer?	Viljandi	11 plants	13 plants and 9 medicines	6 plants and 10 medicines	
Dietrich Timotheus (1859–1929)	Vodka master, manor keeper, and industrial worker	Jõhvi		9 plants		H II 7, pages: 617–702 and 715–716/1889
Jaan Bergmann (1856–1916)	Pastor, translator, and poet	Tartu		2 plants		
Christjan (Kristjan) Koppel (1866–1930)	Medical student; from 1897, Khabarovsk District doctor and civil servant of the Russian Empire; later Consul of the Republic of Estonia (1920–1922); from 1922, a ward doctor in Estonia	Tartu		8 plants		
H. Pärtel (?–?)		Taurida Governorate ^2^		1 plant		
Hans Kosesson (1870–1944)	Assistant to the manor’s vodka master	Tarvastu		30 plants and 30 medicines		H I 3, pages: 261–268 and 297–316/1892
Jaan Miländer (1866–1940)	Medical student; later professor of medical sciences at the University of Tartu and head of the women’s clinic	Saarde		10 plants		
Helene Maasen (1869–1933) (F)	Elementary school teacher and journalist	Palamuse		15 plants		H II 27, pages: 255–278; H III 8, pages: 387–414 and 439–454; H III 15, pages: 143–154; E, pages: 38788–38794, 52232, and 52271/1888–1892
Jaak Kiwisäk (1868–1903)	Farmer; died tragically, shot by poachers	Karksi			3 plants	
Jaan Ostrow (1869–1919)	Manor keeper and promoter of fish farming	Rõngu			9 plants	
Elise Aun (1863–1932) (F)	Writer and poetess	Torma			33 plants and 14 medicines	

^1^: most likely Jakob (b. 1861), as he was an active correspondent of Hurt, although he did not send him any information on plant use. His brother, Jaan Orraw (b. 1852), sent Hurt (from the Vitebsk Province of Russia, where he had settled to live in the early 1880s) 7 plant uses in 1888 (H I 2, pages 561–574), none of which match the information in the Ostrov collection. Both brothers worked as farmers and potters. ^2^: Many Estonians migrated to the Crimean Peninsula at that time and ordered Estonian newspapers from there. ^3^: H. Karu sent Eisen a list of farm names in his municipality in 1892 (E, 1447–1449). Hans Karu (1870–1926) comes from this municipality, but it is not certain that he is the same person. Each “plant” (sample) sent was accompanied by a use. By “medicine”, Ostrov meant either a text on a medicinal plant without an herbarium sample or a non-plant medicine. (F): female correspondent.

**Table 2:** Uses of medicinal plants collected by Ostrov during his appeals and a comparison with other sources (if the plant/use is present).

Family	Taxa	MO Identification	Local Name	UR	Application (Local Name of Disease)	Uses in the Rest of HERBA (19th–20th Centuries)	Uses in Rosenplänter 1830s	Uses in Luce 1829	Uses in Alksnis 1894
Acoraceae	*Acorus calamus* L.	*Acorus calamus*	kalmus	2	Alcohol infusion of roots was drunk against stomach-ache (*kõhuvalu*);Fresh leaves distributed in room against fleas	Widespread Widespread			Use not specified
Amaryllidaceae	*Allium cepa* L.	*Allium cepa*	sibul	2	Bulbs eaten raw with honey against Anthrax (*villitõbi*); Bulbs roasted and juice drunk against lung disease (*rinnahaigus*)	Widespread, but not exact use Widespread		Applied roasted	Cough
Apiaceae	*Angelica sylvestris* L.	*Archangelica officinalis*	heinputk	5	Decoction of roots against urination problems (*ei saa kusta*) and internal problems (*sisikonna kinnitamiseks*); Fresh root chewed against infectious diseases (*külgehakkava tõbi*); Powder made of dried roots ingested against anxiety	Jõhvi parish* in 1937 - -			
	*Carum carvi* L.	*Carum carvi*	köömel	2	Strong alcohol infusion of seeds drunk against stomach-ache (*kinnine kõhuvalu*); Water infusion of flowers or seeds used against flatulence (*kõhu kobisemine*)	Widespread Widespread	Mixed with beer		Other uses
	*Cicuta virosa* L.	*Cicuta virosa*	mürkhain, mürk	4	Whole plant (including roots and leaves) is crushed with salt and applied on erysipelas (*roos*) or tumors (*kasuva*); Baked root is applied on boils (*paised*); Applied on abscess (umbe) when black blood appeared under the skin	- - -			Similar uses
	*Levisticum officinale* W.D.J.Koch	*Levisticum officinale*	lääbus	1	Leaves, stems, and some of the root crushed with a little butter and applied on closed boils (*umbes, üles aand paistetus*)	Other uses		Mixed with manure	
Asphodelaceae	*Aloe arborescens* Mill.		aloe, aloe lill	2	Sap applied on cracked lips; Split leaves applied on burns	- Widespread			Use not specified
Asteraceae	*Achillea millefolium* L.	*Achillea millefolium*	raudriarohi, raudrohi, verihain,	20	Decoction of herbs is a component of epilepsy (*langetõbi*) treatment; Inflorescence decoction against cold, pneumonia and cough, constipation, diarrhoea, excessive bleeding during menstruation, *pistja*-some sharp pain inside the body of unknown origin; Crushed leaves applied to wounds to heal and stop bleeding; Applied also on boils	- Widespread - Widespread Widespread Widespread Widespread Widespread	Present, but no similar uses	Wound plaster	Cough, tuberculosis Homeostasis
	*Antennaria dioica* (L.) Gaertn.	*Gnaphalium dioicum*	kassikäpp	1	Aerial parts boiled along with [*Trifolium montanum*] and drunk against endometritis (*valged*)	Widespread	Present, but no similar uses		
	*Arctium tomentosum* Mill.	*Lappa tomentosa*	takjas, takkäs	4	Roots ground into flour and digested with water against lung disease (*rinnahaigus*); Seeds ingested whole or ground against sharp pain (*pistja*); Juice of leaves applied on wounds	Common in earlier texts Widespread -			Other uses
	*Artemisia absinthium* L.	*Artemisia absinthium*	koihein, koirohi	3	Alcohol infusion of fresh herbs or only leaves used to treat stomach-ache and diarrhoea	Widespread		Similar use among other uses	Similar use among other uses
	*Artemisia vulgaris* L.	*Artemisia vulgaris*	puju, pojokesed	2	Decoction of roots or powdered roots ingested to treat epilepsy (*langetõbi*)	Widespread	Present, but no similar uses		Same use
	*Inula helenium* L.	*Inula helenium*	alandi juur	1	Roots powdered and mixed with butter or grease applied on scabies (*sügelised*)	Other uses			
	*Leucanthemum vulgare* L.	*Chrysanthemum leucanthemum*	arnikas	1	Inflorescence tea against straining (*venitus*)-disease obtained from too much hard work	Widespread (name-based), all plants used resemble *Arnica montana*		*Arnica montana* is present	Other uses
	*Matricaria chamomilla* L.	*Matricaria chamomilla*	kamelid, kummel, kummelid, obinahein, ubinhain	9	Inflorescence tea for women in labour, stomach-ache, diarrhoea, lung disease (*rinnahaigus*), cold and cough; Sap of herb applied on boils (*paised*)	Widespread all (similar) uses	Other uses	Uses unspecified	Other uses
	*Tanacetum vulgare* L.	*Tanacetum vulgare*	reinvarred	1	Aerial parts boiled against chest pain (*rindealt valu*)	Other uses		Other use	Other use
	*Taraxacum officinale* F.H.Wigg. (coll.)	*Taraxacum officinale*	võilill	4	Inflorescences dried, powdered, mixed with alcohol, and ingested against severe diarrhoea (*kõhutõbi*); Teaspoon of sap used to cure constipation; Powdered roots used against jaundice (*kollatõbi*); Aerial parts boiled if a pregnant woman gets hurt and given to her to drink	Widespread Tea widespread Widespread -		Other uses	Other uses
	*Tripleurospermum inodorum* (L.) Sch.Bip.	*Chrysanthemum inodorum*	krambirohi	1	Decoction of aerial parts drunk and used as bath against spasms (*krambid*)	Widespread (name-based)			
	*Tussilago farfara* L.	*Tussilago farfara*	paiseleht	6	Fresh leaves applied on boils; Tea made from dried leaves drunk against lung disease (*rinnahaigus*)	Widespread both uses		Same use	
Betulaceae	*Betula* spp.		[(sauna) viht], [tökat]	3	Powder made from dried birch whisk leaves and mixed with fresh milk cream applied on scabies (*sügelised*); Birch whisk leaves stuffed into the pillow against headache; Birch tar lubricated on the scabies	- Widespread, but not exact use -		Other uses	Other uses
Boraginaceae	*Anchusa officinalis* L.	*Anchusa officinalis*	villirohi	1	Crushed fresh plant applied on anthrax (*vill*) lesions	Widespread (name-based)			
	*Echium vulgare* L.	*Echium vulgare*	roosirohi	1	Crushed fresh plant applied on erysipelas (*roos*) lesions	Widespread (name-based)			
Brassicaceae	*Armoracia rusticana* P.Gaertn., B.Mey. & Scherb.	*Armoracia rusticana*	mädarõigas	1	Holding a small piece of root in one’s mouth believed to heal tuberculosis (*tiisikus*)	Common, but not exact use	Present, but no similar uses		Other use
	*Brassica oleracea var. capitata L.*		hapud kapsad	1	Sauerkraut wrapped on the forehead against headache (*peavalu*)	Widespread			Same uses
	*Capsella bursa-pastoris* (L.) Medik.	*Capsella bursa pastoris*	silmarohi	1	Whole plant boiled in closed vessel and diseased eyes washed (*haiged silmad*) with this water	Widespread (name-based)			
	*Raphanus raphanistrum* L.	*Raphanus raphanistrum*	reigas	1	Fresh root grated and applied on the neck against angina (*kaelahaigus*)	Other uses			Other use
Caprifoliaceae	*Valeriana officinalis* L.	*Valeriana officinalis*	baldrian, jungver, südamevalurohi, rinnarohi	10	Powdered roots ingested with water against cold, malaria (*halltõbi*), arthritis (*luuvalu*), and headache, or mixed with *heinputk* root powder against diarrhoea; Decoction of flowers or roots given to women in childbirth as pain relief; Tea made from leaves and flowers drunk against cough, developing tuberculosis (*tiisikus*); Tea made from leaves and flowers drunk against chest pain (*südamevalu*)	- Common, but not exact use - Widespread (name-based) Widespread (name-based)		Childbirth	Cough, many other uses
Caryophyllaceae	*Silene vulgaris* (Moench) Garcke	*Silene inflate* [Ostrov’s note: “*Silene inflata* probably, but it may also be *Silene nutans”*]	põierohi	1	Additive to medicine to treat epilepsy, part of the decoction of flowering herbs collected before Midsummer’s Day	Other uses		Use not specified	Other use
Cupressaceae	*Juniperus communis* L.		kadakas	2	Twigs boiled and added to bath to treat swollen legs (*paistetanud jalad*), in addition a tea made from pseudo-fruits drunk; Powdered pseudo-fruits mixed with butter and gunpowder applied on scabies (*sügelised*)	Widespread Widespread, but not exact use			
Ericaceae	*Andromeda polifolia* L.	*Andromeda polifolia*	soosassaparillad	1	Decoction of herbs drunk against rheumatism	Widespread			
	*Ledum palustre* L.	*Ledum palustre*	sookaislad	1	Tea made from flowers drunk against tuberculosis (*tiisikus*)	Widespread		Other uses	Same and similar uses
Fabaceae	*Pisum sativum* L.		hernes	1	Boiled with wax against rectal prolapse in children (*kui lapsel “ihu väljas käib”*)	Other uses			
	*Trifolium montanum* L.	*Trifolium montanum*	maarjaristikhein, rohulill, valge ristikhain, valged nupud	6	Aerial parts boiled alone or with [*Antenaria dioica*] and drunk against endometritis (*valged*); Given to a woman during childbirth to maintain strength	Widespread (name-based) -			
	*Trifolium spadiceum* L.	*Trifolium spadiceum*	põldhumalad, rinnatee	2	Decoction of flowers and stems drunk against cough and lung disease (*rinnahaigus*).	Widespread (name-based)			
Lamiaceae	*Glechoma hederacea* L.	*Glechoma hederacea*	jooksva rohi, kassiratas, maalishein, paistus hain	9	Decoction of dried aerial parts drunk against rheumatism (*jooksva*); Decoction used to wash different forms of eczema (*mailased*); Leaves and stems ground with *Urtica urens*, applied with a linen cloth on scabs and herpes (*uhatand*) lesions; Decoction drunk to treat oedema or mixed with *Solanum dulcamara* decoction to wash scabs (kärnad) and edema (*paistes*) swellings	All uses widespread (name-based)		Skin inflammation	Other uses
	*Lamium album* L.	*Lamium album*	naestenõges piimanõges, ema nõges, malaise hein	3	Infertile women whisked in sauna with whisks made from aerial parts to become fertile; Decoction of herbs applied externally to eczema (*mailased*) rashes and also drunk to treat the same; Decoction of flowers used to wash eyes against eye diseases	All uses widespread (name-based)			Leukorrhea
	*Mentha arvensis* L.	*Mentha arvensis*	mündid, vesimünt	2	Tea made from herbs used to treat diarrhoea and stomach-ache	Widespread			
	*Mentha spicata* L.	*Mentha crispa*	münt	2	Tea made from herbs used to treat cold and cough	Widespread			Cough and diarrhoea
	*Thymus serpyllum* L.	*Thymus serpyllum*	kolme-korralised hainad, liivatee	2	Treat an incomprehensible disease (*arusaamata tõbi*) and sudden diseases (*rabandus*)	Widespread		Other use	
Linaceae	*Linum usitatissimum* L.		[linane riie]	4	Linen cloth used to cover different medicines in treating boils and eczema; Scraps or ashes of linen cloth used to stop bleeding	Widespread -			Other uses
Menyanthaceae	*Menyanthes trifoliata* L.	*Menyanthes trifoliata*	ubaleht	7	Strong alcohol infusion of sun-dried leaves, left overnight in bread-stove, used to treat tuberculosis (*tiisikus*) and lung disease (*kuivtõbi*); Decoction of leaves, two spoonfuls ingested every two hours to treat fever associated with cold (*külmapalavik*), stomach diseases (*kõhutõbi*), edema (*vesitõbi*); Take a small amount of dried stem powder against constipation	Widespread use, but not the specific preparation Common, but not exact preparation - Similar use from Kuusalu (1964)	Cough		Tuberculosis, cough, fewer, cramps, oedema, and other uses
Orchidaceae	*Dactylorhiza maculata* (L.) Soó	*Orchis maculata*	jumalakäpp	2	Powder of dried flowers and roots given against sudden diseases (*rabandus*) and the sudden onset of other diseases, usually associated with witchcraft (*äkkiline haigus*)	Other uses			Other uses
Parmeliaceae	*Cetraria islandica* (L.) Ach.	*Lichen islandicus*	põdrasammal	1	Boiled until the water becomes jellied and ingested against cough	Widespread			
Pinaceae	*Picea abies* (L.) H.Karst.		kuusk, [vaik/tõrv]	4	White part of the bark held between the lips to heal herpes; Fresh resin applied on boils and old wounds directly or covered with butter or boiled with rye shoots, sour cream, and grease	- Similar uses widespread			Other use
	*Pinus sylvestris* L.		mänd	1	Needles used to make a bath against rheumatism (*jooksva*)	Similar uses widespread			Other uses
Plantaginaceae	*Plantago major* L.	*Plantago major*	teeleht	15	Rubbed leaves applied on boils, and old and fresh wounds; Sap of leaves given against vomiting blood and frequent menstruation; Seeds are ingested in case of diarrhea and the risk of premature birth	Widespread Similar use found in Iisaku (1929) A few similar uses Widespread -	Wounds	Ulcers	Diarrhoea, dysentery, emerging ulcer
	*Veronica officinalis* L.	*Veronica officinalis*	maaleserohi	2	Boiled and washed with that water (usually with *Viola tricolor* L.); Decoction of herbs drunk against different forms of urticaria (*maa-alused*)	Widespread (name-based)	Similar use and name	Eczema and other uses	
Poaceae	*Briza media* L.	*Briza media*	külmaväristuserohud, värisajahein	2	Decoction of herbs drunk against malaria (*külmatõbi*)	Widespread (name-based)	Similar use and name		Other uses
	*Secale cereale* L.		rukis	1	Young shoots boiled with fresh spruce resin, sour cream, and grease applied on wounds directly	Similar uses widespread			Other uses
Polygalaceae	*Polygala amarella* Crantz	*Polygala amara*	emakajuur, jooksvakaetus, kaitused, kõõmahein, naeste päästja	5	Decoction of herbs drunk against lung disease (*rinnahaigus*), tuberculosis (*tiisikus*), and rheumatism (*jooksva*) or used to wash face (after sunset) to treat mouth scurf (*suu kõõm*); Dried and boiled, given to woman to drink with sugar before childbirth to ease giving birth	All widespread (name-based) -			Other use
Rosaceae	*Fragaria vesca* L.	*Fragaria vesca*	maasikas	4	Tea made from dried leaves and flowers drunk against cough	Widespread			Cough and other uses
	*Geum rivale* L.	*Geum rivale*	härjapää, karukollad	2	Decoction of leaves and inflorescences drunk against rheumatism (*jooksva*); Decoction of inflorescences drunk to induce sweating in case of cold	- Common (name-based)			
	*Geum urbanum* L.	*Geum urbanum*	laste kõhurohi, kõhutõbe juured	2	Decoction of roots and leaves drunk against diarrhoea and stomach-ache, especially in children	Common (name-based)			Other use
	*Potentilla erecta* (L.) Raeusch.	*Tormentilla erecta*	tedremadar, nabahain, tedremaranas	7	Alcoholic infusion of roots used against diarrhoea and stomach-ache; Powdered dry roots ingested with water against stomach-ache	All uses widespread			Similar uses
	*Rosa canina* s.l.	*Rosa canina*	kibuvits	1	Tea made from petals used against cough	*Rosa* spp. not differentiated, use of *Rosa* widespread			
	*Sorbus aucuparia* L.		pihlakas	1	Decoction of bark used against headache	Other uses			Other uses
Scrophulariaceae	*Verbascum thapsus* L.	*Verbascum thapsus*	vägihein, üheksamehevägi, üdismed	8	Fresh leaves applied on wounds; Rubbed leaves applied on inflammations, especially between the toes; Tea made from flowers drunk against tuberculosis (*tiisikus*)	All uses widespread		Similar uses	Other uses
Solanaceae	*Nicotiana rustica* L.		tubakas	2	Dried leaves applied on snakebites	Widespread		Other uses	Other uses
	*Solanum dulcamara* L.	*Solanum dulcamara*	maavits, majakad, solknamarjad, solknavarred	8	Seeds eaten against internal parasites of the *Ascarida* family (*solknad*); Tea used against internal pain (*seestvalu*) and edema (*vesitõbi*); Boiled with *Glechoma hederacea* and the decoction used to wash swellings (edema) and scabs, but also drunk against internal pain (*seesthaigus, seestvalu*) and different forms of urticaria (*maa-alused*)	widespread (name-based) - Widespread - Widespread (name-based)	Similar use and name	Large roundworm infestation	Other uses
	*Solanum tuberosum* L.	*Solanum tuberosum*	kartul, kartohvel	3	Bulb scrapings applied on inflamed areas; Starch mixed with *Urtica urens* seeds and ingested against diarrhoea	Widespread Common, but not exact preparation			Other uses
Urticaceae	*Urtica dioica* L.	*Urtica dioica*	nõges	1	Dried leaves smoked against tuberculosis (*tiisikus*)	Common			
	*Urtica urens* L.	*Urtica urens*	raudnõges	7	Seeds mixed with potato flour and ingested against diarrhea; Leaves rubbed against cheek until the toothache is gone; Seeds ingested with alcohol against malaria (*halltõbi*); Decoction of herbs used in bath to treat urticaria; Whisked in sauna with fresh herb against sudden diseases (*rabandus*); Decoction of herbs used to bathe someone with chickenpox (*tuulerõuged*)	Common, but not exact prep. Common, but not exact prep. Widespread Common (name-based) - -			Other uses
Violaceae	*Viola canina* L.	*Viola canina*	seakapsas	1	Sap used to heal fresh wounds	-			
	*Viola palustris* L.	*Viola palustris*	südamevalurohi	1	Decoction of leaves against heart pain (*südamevalu*)	Widespread (name-based)			
	*Viola tricolor* L.	*Viola tricolor*	kesalill, maaaluse rohi, mailaseroht	6	Medicine for 9 diseases; Decoction of dried herbs is used to wash different forms of urticarial (*maa-alused, mailased*) rashes especially in children, but a little of it also drunk (boiled with *Veronica officinalis* L.); Decoction of herbs drunk against rheumatism (*jooksva*) and tuberculosis (*tiisikus*)	All uses widespread (name-based)		Eczema	Cough

## Data Availability

EKS (=EKnS, Eesti Kirjanduse Seltsi rahvaluulekogu), H (Jakob Hurda rahvaluulekogu), E (Matthias Johann Eiseni rahvaluulekogu). Located at the Estonian Folklore Archives at Estonian Literary Museum, in Tartu, Estonia.
